# Deciphering the Chemical Footprint of Renewables

**DOI:** 10.1021/acs.est.5c17593

**Published:** 2026-08-04

**Authors:** Tasneem Tawalbeh, Zhanyun Wang, Kathrin Fenner, Yuxin Wang

**Affiliations:** † Systems Science and Industrial Engineering, 14787 Binghamton University, 440 Vestal Pkwy E, Binghamton, New York 13902, United States; ‡ Department of Green Technology & Danish Institute for Advanced Study (DIAS), University of Southern Denmark, 5230 Odense, Denmark; § Empa–Swiss Federal Laboratories for Materials Science and Technology, Technology and Society Laboratory, 9014 St. Gallen, Switzerland; ⊥ National Centre of Competence in Research (NCCR) Catalysis, 8093 Zürich, Switzerland; # Department of Environmental Chemistry, Swiss Federal Institute of Aquatic Science and Technology (Eawag), 8600 Dübendorf, Switzerland; ¶ Department of Chemistry, University of Zürich, 8057 Zürich, Switzerland; ▽ Systems Science and Industrial Engineering, Binghamton University, Binghamton, New York 13902, United States

**Keywords:** renewable energy, chemical footprint, life cycle impacts, hazardous substances

## Abstract

Renewable energy technologies are expected to see substantial growth in development and deployment for combating climate change. However, their current manufacturing, deployment, operation, and end-of-life management involve many hazardous chemicals, which can be released at various stages of the life cycle, posing significant risks to human health and the environment, and potentially undermining the overall sustainability benefits of renewable energy technologies. Given the long lifespan of renewable energy technology infrastructure, such chemical impacts may persist for decades, underscoring the need to urgently address both existing and future risks throughout the life cycles of various technologies as deployment scales up. Building on emerging evidence across renewable technologies, we emphasize that their environmental benefits should be achieved while minimizing risks to human and ecosystem health. To foster a sustainable energy transition, greater attention is warranted toward the embedded chemical footprint. This includes improving the measurements, transparency, and early consideration of chemical impacts in renewable technology development, and identifying and addressing chemical impact hotspots across existing technologies. Achieving this requires collaborative efforts across renewable energy and energy storage sectors and beyond.

## Graphical abstract

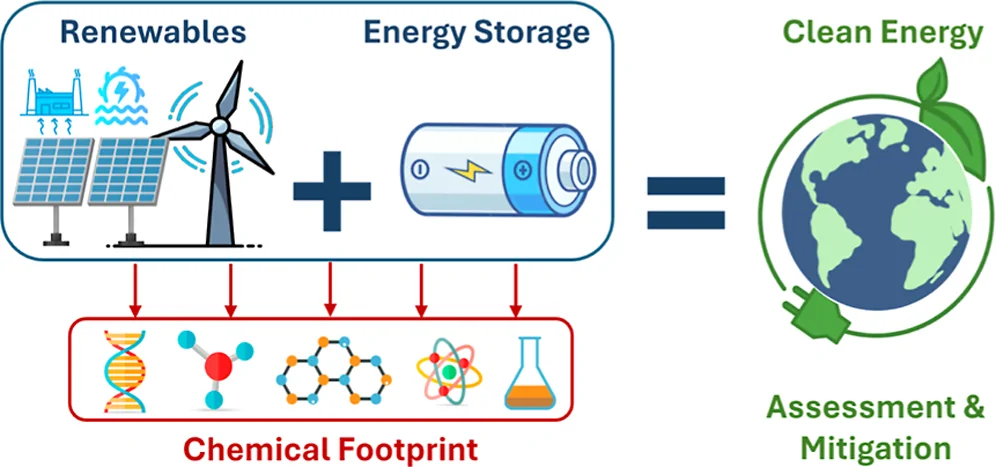

## Introduction

Humanity is facing the triple planetary crisis of climate change, biodiversity loss, and pollution. Renewable energy plays a critical role in mitigating climate change, reducing greenhouse gas emissions and co-emitted air pollutants. The current primary sources of renewable energy are solar, wind, hydropower, biomass, geothermal power, and fuel cells, all of which are expected to experience significant expansion in the coming years (Figure 1).^1^

**Figure 1:**
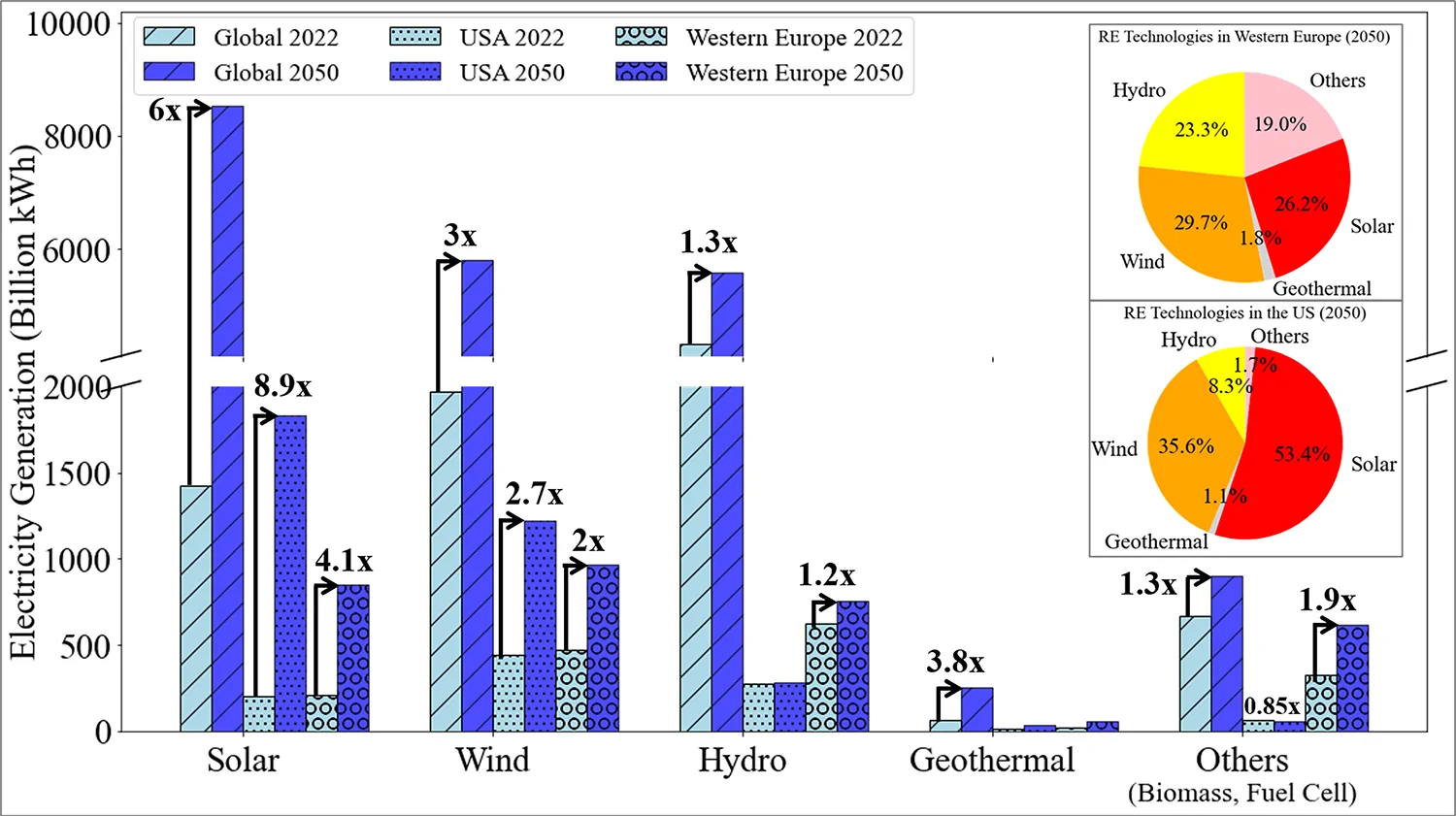
Electricity generation from renewable technologies in 2022 and expansions projected by 2050 at the global, U.S., and Western European levels.^1^ Detailed data and citations are listed in Table S1.

A large body of research has evaluated renewable energy technologies across multiple environmental impact categories (e.g., air pollution, resource depletion); among them, carbon-related metrics dominate policy priorities and decision-making. However, renewable technologies are not chemically neutral. Their current manufacturing, deployment, operation, and end-of-life (EOL) management involve many hazardous chemicals, whose releases may lead to severe environmental pollution and damage on human and ecosystem health. For example, despite its well-known toxicity to the human nervous, reproductive, and developmental systems, lead is still widely used in traditional silicon solar cells and next-generation perovskite solar cells (PSCs), with documented cases of environmental pollution resulting from leaching.^2,3^ Another example is lithium-ion batteries (LIBs). While they offer an effective energy storage solution, their production, disposal, and recycling have been associated with the environmental release of per- and polyfluoroalkyl substances (PFASs) and other hazardous chemicals,^4^ as well as broader ecological concerns such as land degradation and habitat loss resulting from lithium mining.

Although studies are emerging to explore the intersections of climate change mitigation, industrial manufacturing, and chemical pollution,^5^ they often focus on a limited set of well-known chemicals, particularly greenhouse gases^6^ (e.g., CO_2_ and CH_4_) and air pollutants (e.g., SO_2_ and NO_*x*_).^7^ As a result, the broader diversity of chemicals embedded in renewable energy technologies and their implications have received far less attention than their climate impacts. Similar discussions about “embedded toxicity” have recently emerged in the building sector, where the majority of sustainability assessments have emphasized upfront greenhouse gas emissions associated with building materials while limited attention has been paid to chemical impacts.^8^

Furthermore, even when chemical impacts were considered, existing studies provided only limited consideration of emerging contaminants (e.g., certain PFASs and specific transformation products) and insufficient coverage of life cycle stages such as disposal, recycling, degradation, and other EOL processes.

Here, we aim to draw attention to the embedded chemical footprint and emphasize the importance of identifying and understanding hot- and blind-spots of chemical releases, exposure, and impacts throughout the life cycle of renewable energy technologies. Overlooking these chemical dimensions may undermine the overall climate benefits of renewable technologies. Our intent is not to propose a new assessment framework, but call for systematically integrating chemical considerations into renewable energy sectors. Robust methodological frameworks such as life cycle assessment (LCA) and chemical risk assessment have been established for quantifying various environmental impacts across product life cycles and have been widely applied to energy systems.^9–11^ However, a more systematic understanding of, and inventories for, chemical presence, degradation pathways, release potential, and EOL emissions are still needed before these impacts can be comprehensively quantified using existing assessment frameworks. In other words, renewable energy technologies provide clear environmental benefits, particularly given their essential role in combating climate change, but they may also involve trade-offs across different impact categories. Drawing attention to their embedded chemical footprint may help guide improved assessment and management of chemical risks in the renewable energy production and storage sectors, including the continued refinement and application of LCA frameworks.

In this Perspective, we first provide a concise overview of known hazardous chemicals associated with major renewable technologies to underscore the scale and urgency of addressing the often-overlooked impacts. We then explore pathways to better integrate the embedded chemical footprints in the development and management of future energy systems.

## Chemical Footprints Embedded in Renewables

Chemical substances, including functional materials, processing mixtures, and specific compounds, play a dual role in the renewable energy sector, contributing to the technology advancement while also posing risks to the environment and human health. To help draw attention to these often-overlooked dimensions of renewable energy systems, we use the term “embedded chemical footprint” to describe the presence, use, release, transformation, and potential impacts of chemical substances across all life stages of energy technologies, including production, operation, and EOL. Similar to discussions surrounding “embedded carbon” and “embedded toxicity,” the term “embedded chemical footprint” emphasizes the importance of systematically collecting information on the ecosystem and human health impacts of chemicals used throughout the production, operation, and EOL management of renewables and associated energy storage systems. Given that solar energy is expected to dominate the renewable energy sector (Figure 1), we focus in some details on the chemical footprint of solar technologies and their associated energy storage (battery) technologies during the ongoing energy transition. Figure 2 provides a qualitative overview of potential chemical release pathways. The relative magnitude and likelihood of these emissions may vary substantially depending on technology design, operating conditions, and management practices, which remain insufficiently characterized at present.

**Figure 2:**
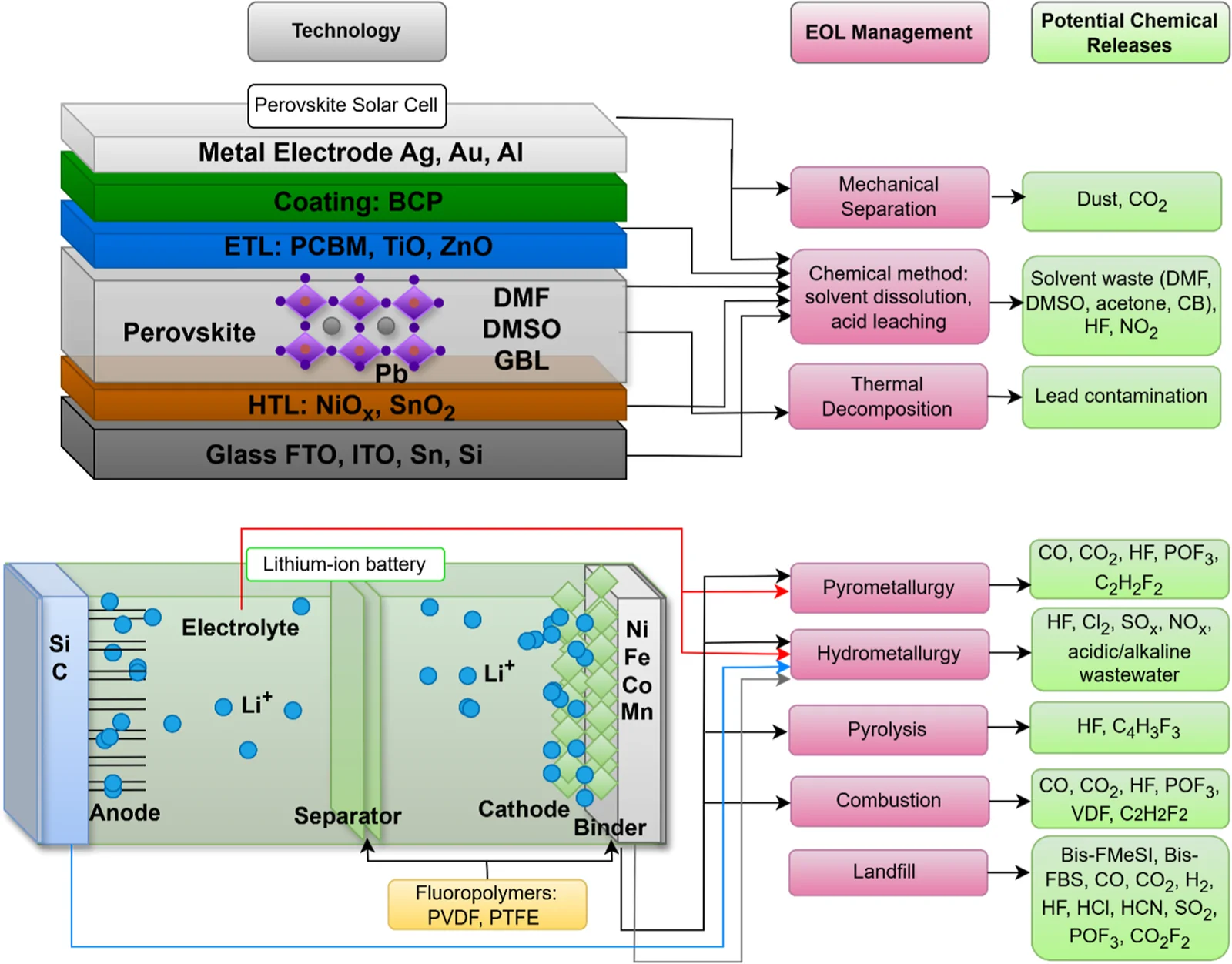
Chemicals used in perovskite solar cells and lithium-ion batteries, and their EOL management options and associated (potential) chemical releases. Colored arrows denote different material pathways: red = whole battery recycling pathways; blue = anode materials; black = fluoropolymer-containing components, such as separator and binder; gray = cathode materials. **Abbreviations:** FEP—Fluorinated ethylene propylene; ETFE—Ethylene tetrafluoroethylene copolymer; PVDF—Polyvinylidene difluoride; PVF—Polyvinyl fluoride; PET—Polyethylene terephthalate; FEVE—Fluoroethylene vinyl ether; BCP—Bathocuproine; DMF—Dimethylformamide, DMSO—Dimethyl sulfoxide; GBL—Gamma-butyrolactone; CB—Chlorobenzene; FTO—Fluorine-doped tin oxide; ITO—Indium tin oxide; PTFE—Polytetrafluoroethylene; VDF—Vinylidene fluoride (monomer); bis-FMESI—bis­(perfluoromethane) sulfonimide; and bis-FBSI—bis­(perfluorobutane) sulfonimide. Arrow colors indicate different material pathways: red arrows denote the treatment of whole spent lithium-ion batteries via major recycling processes (pyrometallurgy and hydrometallurgy); blue arrows denote anode components; gray arrows denote cathode components; and black arrows denote fluoropolymer-containing components (e.g., PVDF binders and separators) entering these processes.

### Solar Panels

Solar panels have a significant chemical footprint, primarily due to their production processes,^12^ which involve the use of many hazardous chemicals that are toxic or combustible,^13^ particularly toxic metals such as lead, cadmium, and nickel (Figure 2).^2^ Lead remains a cornerstone of PSCs performance, although per-unit content is three times less than in silicon panels.^14^ PSCs have been the focus of next-generation solar technologies as they rival silicon in performance and will significantly reduce the cost of solar energy.^15^ Damaged or degraded solar cells due to hail, tornadoes, or operation have been reported to leach lead at levels of 1–10 mg/L.^2,3^ These concentrations are 50- to 600-fold higher^16^ than the United States Environmental Protection Agency (U.S. EPA)’s action level for lead in drinking water (0.015 mg/L).^3^ The reported concentrations are typically derived from controlled or worst-case experimental scenarios, which may not directly reflect environmental concentrations under real-world conditions where release pathways and sites specific conditions could influence exposure. Additionally, there are currently no cost-effective methods for lead management at the disposal stage, especially for PSCs,^17^ which underscores the importance of mitigation strategies during EOL management. At EOL, lead can be released into the environment, especially as a majority of spent silicon panels are landfilled outside the European Union (EU).^17^ Given that the cumulative mass of spent solar modules is projected to reach 80 Megatonnes (i.e., corresponding to 19,704–78,818 tonnes of incorporated lead) globally by 2025,^18^ effective monitoring and management of lead during both operation and disposal are urgently needed to safeguard the ecosystem and human health.^17^

Moreover, nearly every step in PSC manufacturing requires large amounts of organic solvents, such as chlorobenzene, dichlorobenzene (DCB), dimethylformamide (DMF), and *N*-methylpyrrolidone (NMP) to facilitate deposition and crystallization of the layers.^19^ While this solvent-based processing method offers significant advantages for large-scale fabrication on both rigid and flexible substrates, it also introduces significant environmental pollution risks.^20^ As of December 2023, the production of DMF has been highly restricted in the EU due to its classification as a reproductive toxicant.^21^ Similarly, the solvent chlorobenzene is regulated by the U.S. EPA with a threshold limit of 0.1 mg/L in drinking water due to its liver, kidney, and nervous system damages.^3^ Major potential emission sources of solvents during solar cell manufacturing including transportation, storage, and industrial wastewater discharges, together with flammability risks, pose threats of contamination and exposure to both the environment and human health.^22^

PFASs are another group of chemicals widely applied in the renewable energy sector because of their exceptional properties, such as chemical and thermal stability, and water- and oil-repellency. Uses of PFASs, including many fluoropolymers, have been reported in solar collectors, photovoltaic cells,^23^ wind turbine blades,^4^ and various battery compositions.^24,25^ PFASs are commonly known as “forever chemicals” due to their extreme persistence in the environment. Exposure to perfluorooctanoic acid (PFOA) and perfluorooctanesulfonic acid (PFOS) has been associated with various harmful health effects, including impaired immune system, cancer, liver and kidney diseases, and reproduction and developmental complications.^26^ The predominant PFASs used in solar modules are fluoropolymers, which serve as protective barriers to mitigate moisture ingress and environmental degradation.^27^ For example, polyvinylidene fluoride (PVDF), commercially known as Tedlar^TM^, is extensively used in the backsheet films of crystalline silicon panels,^27^ comprising about 1.5 mass % of the panel and serving to protect the silicon cells while providing insulation.^28,29^ While the use of high-molecular-weight fluoropolymers in these applications may be considered safe under normal conditions, other stages of their life cycle, such as production, disposal, and degradation, may pose significant risks to the environment and human health (see Box [Boxed-text dbox1]).^30^ Additionally, side-chain fluorinated polymers (Supporting Information text 1.1) may contain low-molecular-weight PFASs as impurities, or break down and form low-molecular-weight PFASs, and thus release them into the environment along the life cycle.^31^ Indeed, low levels of perfluorohexanoic acid (PFHxA)—a common impurity and degradation product of 6:2 fluorotelomer-based side-chain fluorinated polymers, and currently restricted in the EU^31,32^ have been detected on eight bifacial solar panels from different producers during their use phase.^33^

1 **The Environmental impacts throughout the life cycle of fluoropolymers.**

Fluoropolymers, including polytetrafluoroethylene (PTFE), PVDF, and their copolymers PVDF-trifluoroethylene, PVDF*-co-*hexafluoropropylene (HFP), and PVDF*-co-*chlorotrifluoroethylene (Figure S1), possess outstanding thermal, electrical, and chemical stability, leading to widespread use in the field of renewable energy technologies.^29^ The production of fluoropolymers and their precursors involves and releases various PFASs and other fluorinated chemicals, resulting in human exposure through air, water, and food.^34^ The infamous examples are the salts of perfluoroalkyl carboxylic acids (PFCAs) such as PFOA, which were the most widely used processing aids during the polymerization processes of many fluoropolymer products, including different grades of PTFE and PVDF.^35^ Historically, fluoropolymer production has been identified as the dominant source of PFCA emissions, accounting for approximately 80% of global releases between 1951 and 2002 based on industry-wide estimates.^35^ These releases have led to significant contamination of downstream environments, contributing to much increased health burdens, including elevated cancer rates, which have prompted numerous lawsuits in the U.S. and other regions.^36^ To date, PFOA has been largely replaced, yet many alternatives are PFAS-based and exhibit similar hazards.^37,38^ Even the nonfluorinated processing aids can generate and release a broad range of unintended fluorinated byproducts.^39^ Fluoropolymer production also emits a variety of unreacted substances, including raw materials and intermediates (e.g., chlorodifluoromethane (HCFC-22), trifluoroethane (HFC-143a)), residual reagents, byproducts from monomer synthesis (e.g., trifluoromethane (HFC-23), perfluoroisobutene (PFIB), perfluorocyclobutane (c-C_4_F_8_)), unreacted monomers (e.g., tetrafluoroethylene (TFE), HFP, VDF)), oligomers formed during polymerization, and fluoropolymer particles.^40–42^ Beyond the release of toxic PFASs and other fluorinated compounds, some of these emissions are potent greenhouse gases. For example, emissions of HFC-23 to air—primarily originating from HCFC-22 synthesis, over 80% of which is used as a feedstock for manufacturing TFE and HFP—were estimated at 15,900 ± 900 tonnes in 2018 ^43^ and 17,300 tonnes in 2019 globally.^44^ These emissions are equivalent to over 200 million tonnes of CO_2_-equivalent greenhouse gases.

While valued for their thermal stability, certain fluoropolymers can degrade under operational conditions. Nafion ionomers, for instance, can produce perfluoro­(2-methyl-3-oxa-5-sulfonic pentanoic) acid during fuel cell operation, and the fluorinated ionomers formerly produced by 3M^TM^ can degrade into perfluoro­(4-sulfonic butanoic) acid.^45^ Furthermore, a substantial portion of fluoropolymers may end up in the environment and persist, while another portion may undergo open burning or high-temperature incineration. Open burning may pose significant environmental risks due to uncontrolled emissions of low-molecular-weight PFASs such as various perfluoroalkyl acids (PFAAs).^46–51^ High-temperature incineration (≥850 °C) with strict operational controls and flue gas treatment significantly improves fluoropolymer degradation compared to open burning. But despite some evidence of low atmospheric PFAS emissions at these conditions, the formation, fate, and environmental release of diverse and often unmonitored fluorinated degradation products—including those in flue gas, bottom ash, and fly ash—remain incompletely understood.^51^

### Fuel Cells

Fuel cells are not inherently renewable, but they are considered part of the renewable energy system when powered by hydrogen produced from renewable energy sources such as solar, wind, or biomass. They can also be considered part of energy storage strategies, playing a key role in energy conversion and long-term energy storage coupled with green hydrogen production. Fluoropolymers are key materials in fuel cell electrolyte polymeric membranes.^29^ For example, Nafion—a perfluorinated polymer that contains a PTFE backbone and perfluoroalkylether side chains terminating in sulfonic acid groups^48^ is widely used in fuel cell technologies due to its outstanding proton conductivity and chemical and mechanical stability.^52^ In addition to general environmental impacts during the life cycle of fluoropolymers (Box 1), reprocessing of used Nafion via solvent-based dissolution (e.g., DMSO, DMF, or water/ethanol mixtures)^53^ produces large volumes of corrosive and toxic waste.^54^ Recycling of fuel cell membranes generally requires high-temperature pyrolysis (400–600 °C),^53^ which is insufficient for Nafion mineralization and can release low-molecular-weight PFASs, including PFCAs^48^ and perfluoroalkanesulfonic acids (PFSAs),^55^ contributing to global PFAS pollution. This highlights the need for a better understanding of waste management approaches and the chemical footprints associated with the full life cycle of fuel cell materials, from production to EOL disposal.

### Batteries

Batteries, especially LIBs at this moment, are critical in renewable energy storage, yet their chemical footprint remains largely underrecognized.^4^ Lithium mining generates significant quantities of wastewater, especially from lithium brines, which are highly concentrated in toxic elements, including arsenic and boron and have low pH levels (2.5–4.8). These conditions pose serious ecological and human health risks, particularly in the event of leaks of mining wastewater or spent brines from evaporation ponds, or of direct injection of spent brines into the subsurface.^56^

Fluorinated substances, including PFASs, are also extensively applied in the components such as electrodes, electrolytes, binders, and separators of many LIBs (Figure 2).^24^ For instance, PVDF is the most commonly used binder to attach active electrode materials to the metal foil current collector. To enhance LIB performance at low temperatures, another group of PFASs, lithium bis­(perfluoroalkane) sulfonamides (LiTFSI), has been used as alternatives to the less stable LiPF_6_ electrolytes.^24^ However, emerging evidence indicates bis­(perfluoroalkane) sulfonimides (bis-FASIs) have been found to be released from battery manufacturing facilities, and to impair the swimming velocity of *Daphnia magna* at very low concentrations of below 10 ng/L, potentially linked with neurobehavioral impacts.^4^

Human exposure to PFASs used in batteries primarily occurs during battery manufacturing, landfill disposal, and recycling of batteries.^24^ For example, PFOA and hexafluoropropylene oxide oligomers (e.g., HFPO-TrA) were detected as impurities in PVDF and PTFE materials and products used for battery materials.^24^ The steps of cropping, assembly, and filling may release harmful compounds from electrolyte materials to the workspace, including fluorinated ethers (such as methyl nonafluorobutyl ether), fluorinated carbonates (such as fluoroethylene carbonate/3,3,3-fluoroethylmethyl carbonate/1,1,2,2-tetrafluoroethyl-2′,2′,2′-trifluoroethyl ether), and fluorinated phosphonate additives.^24^ However, existing occupational safety guidelines generally lack specific standards for PFASs,^57^ although some employers implement protective measures based on internal protocols, best practices, and emerging research.^58^

During LIBs operation, battery damage, thermal runaway, explosion, and disposal (Figure 2) can release toxic gases (e.g., CO, SO_2_, H_2_S, HF), polycyclic aromatic hydrocarbons, particulate matters, and PFASs into the environment.^59,60^ A recent experimental study reported 10–60 μg of 22 different PFASs released per kg of LIBs in soot and particulates during thermal runaway, with total PFAS emissions positively correlated with cell temperature.^61^ Among those, perfluorobutanesulfonic acid (PFBS) and perfluorobutanoic acid (PFBA) were detected at the highest concentrations in all eight prismatic lithium–nickel-manganese–cobalt-oxide (NMC) cell tests.^61^ Notably, although PFOA is banned under the Stockholm Convention, it was detected in all eight tests at concentrations ranging from 0.05 to 0.62 ng PFOA per gram of soot.^61^ Furthermore, PFASs used in batteries can be released into the environment during the battery disposal stages, such as recycling and landfilling.^25^ For example, LIBs discarded in landfills contribute to the presence of bis-FMeSI in landfill leachates (Figure 2).^4^ Common LIB recycling methods include pyrometallurgy, hydrometallurgy, and direct recycling. All these technologies primarily focus on recovering valuable metals from LIBs, and therefore often overlook side releases of hazardous compounds such as PFASs, volatile organics, and toxic gases formed during processing into various environmental media.^25,50^ Pyrometallurgy employs high temperatures (up to 1600 °C) to recover metals, decomposing fluorinated binders and electrolytes into volatile species,^25,60^ including CO, CO_2_, H_2_O, HF, and other fluorinated gases^62^ such as POF_3_ and C_2_H_2_F_2_ (Figure 2).^63^ Hydrometallurgy relies on chemical leaching, separation, and recovery of valuable metals using various solvents (e.g., DMF, DMSO, and acids) to dissolve binders and extract metals,^64^ but generates toxic gases (Cl_2_, SO_*x*_, NO_*x*_) and large volumes of acid brine (Figure 2).^65,66^ Direct recycling seeks to regenerate cathode materials while preserving their chemical structure, potentially lowering energy use and reagent costs.^67^ However, it includes labor intensive pretreatment involving mechanical treatment.^68^ Thermal treatment and hydrometallurgical approaches are sometimes necessary to separate and extract useful materials, which might generate comparable chemical footprints.

### Other Renewable Technologies

Given the diversity of renewable energy technologies, we examine solar and battery systems in greater detail as representative examples where chemical use and associated risks are relatively well documented, while acknowledging that similar considerations should be extended to other renewable technologies in future work. While not presented in detail, other technologies also carry overlooked chemical footprints. Studies on the environmental impacts of offshore wind farms have mostly focused on habitat alteration, underwater noise, electromagnetic fields, and fisheries exclusion. However, multiple parts of offshore wind farms can also release hazardous chemicals, including the corrosion protection systems, coatings, galvanic anodes, rotor blades, cables, and grouting materials.^69^ More than 200 organic and inorganic compounds have been identified as potential emissions from offshore wind infrastructure, with coatings and corrosion protection systems representing the dominant and continuous sources.^69^ However, limited occurrence data and incomplete toxicity information continue to constrain the ability to conduct a more comprehensive risk assessment. For example, coatings for offshore wind energy devices may act as a major, continuous source, related to the composition of the polymeric coatings and additives used therein.^70^ Mechanical and chemical degradation processes initiated by oxidation or hydrolysis can break down the polymer structure into small molecules,^69^ causing releases of a wide range of chemical pollutants (e.g., bisphenol A, glycols, toluene diisocyanate, methylene diisocyanate, methylenedianiline, 4-tert-butylphenol, and plasticizers) and particles into the environment.^69^ Despite limited data on chemicals leaching from polyurethane coatings,^69^ a total of 48 chemicals have been identified in aqueous leachates, including, but not limited to, toluenesulfonamide derivates, methylenediisocyante derivates, and toluenediisocyanate derivates.^71^ Degradation processes can also generate additional compounds that may leach into surrounding waters, several of which have been identified in catalogues of prioritized hazardous chemicals.^72^

In addition, galvanic anodes in the offshore wind technology are identified as the second most important source of chemicals, emitting hazardous metals (e.g., cadmium, lead, mercury, and nickel) into the marine environment at low levels.^70^ Specifically, a recent study indicates that offshore wind infrastructure can act as a source of chemical pollution through releases from corrosion protection systems.^73,74^ For example, large-scale deployment of offshore wind farms has been estimated to release substantial quantities of metals (e.g., aluminum, zinc, and indium), with projected emissions expected to increase by an order of magnitude by 2050.^73^ This work represents one of the first efforts to quantify pollutant inputs directly released from offshore wind structures at scale and to assess their potential toxicological implications. The released metals can accumulate in sediments and marine organisms, raising concerns about long-term ecosystem impacts and potential human exposure via seafood consumption, particularly in regions where aquaculture coexists with offshore wind installations. During the use phase, oil, grease, and lubricants (e.g., phenols and hydrocarbons, diesel and naphthalene) represent the largest group of chemicals that may be released during accidental releases,^69^ and may also contain fluorinated compounds.

In addition to the releases of chemicals that are either intentionally used or unintentionally present in renewable energy infrastructure, the deployment of these technologies can also mobilize existing hazardous substances in the environment. For example, geothermal systems can contribute to the chemical footprint of renewable energy through mobilization of naturally occurring pollutants such as arsenic (As). Arsenic-rich geothermal fluids can be released during exploration, energy production, and waste management, posing risks to drinking/irrigation water and food systems, highlighting the need for systematic monitoring of arsenic and implementation of effective mitigation and waste management strategies.^75^ Incorporating such anthropogenically mobilized natural pollutants into life cycle considerations is essential for ensuring the safe and sustainable development of geothermal energy systems. In addition to arsenic, geothermal fluids may contain elevated concentrations of other hazardous elements such as boron, which can exceed hundreds of mg/L.^76^ Improper management of geothermal fluids, including inadequate construction or uncontrolled discharge, can lead to boron contamination of surface waters and soils.^76^ At certain geothermal plant sites (the Wairakei Geothermal Power Station in New Zealand and the Larderello–Travale Geothermal Field in Italy), geothermal steam used for power generation can lead to volatilization of mercury and its release to air via the cooling tower exhaust.^77^ Mercury scrubbers can be used on-site at the geothermal power plant to reduce air emissions, leading, however, to secondary waste that is required to be properly disposed of as hazardous waste. These challenges highlight the importance of incorporating pollutant-specific monitoring and treatment considerations into the life cycle management of geothermal systems.

## Future Energy System Built with Renewables: An Environmental Urgency

The large-scale deployment of solar, wind, and battery systems (Figure 1) is expected to significantly increase the total chemical footprint associated with these systems. One well-studied aspect of this footprint is mineral demand. For instance, the International Energy Agency (IEA) projects a quadrupling of the mineral demand for renewable technologies by 2040.^78,79^ Figure 3 compares the demand for individual selected materials in solar, wind, and battery technologies in 2020 with projections for 2050. It highlights both the variation in resource utilization across technologies and the potential for substantial growth in raw material consumption, serving as an indicator of the significantly increased chemical footprint associated with renewable energy systems. For example, solar modules alone rely on a wide array of raw materials, including silicon, lead, silver, copper, aluminum, tin, and indium, many of which require chemically intensive extraction and purification processes. The annual demand of silicon, aluminum, and copper, all categorized as critical raw materials by the EU, is projected to increase by factors of 2–20, 4–27, and 2–20 from 2020 to 2050, respectively.^80^ Similarly, the expanding demand for wind turbines will significantly increase the need for rare earth elements (REEs), and could potentially outpace the current U.S. domestic supply of nickel, glass fiber, and REEs-containing electrical steel.^81^ Specifically, the demand for REE materials such as dysprosium, neodymium, praseodymium, and terbium used in wind turbines are predicted to increase 7- to 20-fold over the next two decades. Moreover, mineral demands for batteries are forecasted to rise more than 10-fold, with lithium demand alone expected to grow 20-fold.^67^

**Figure 3:**
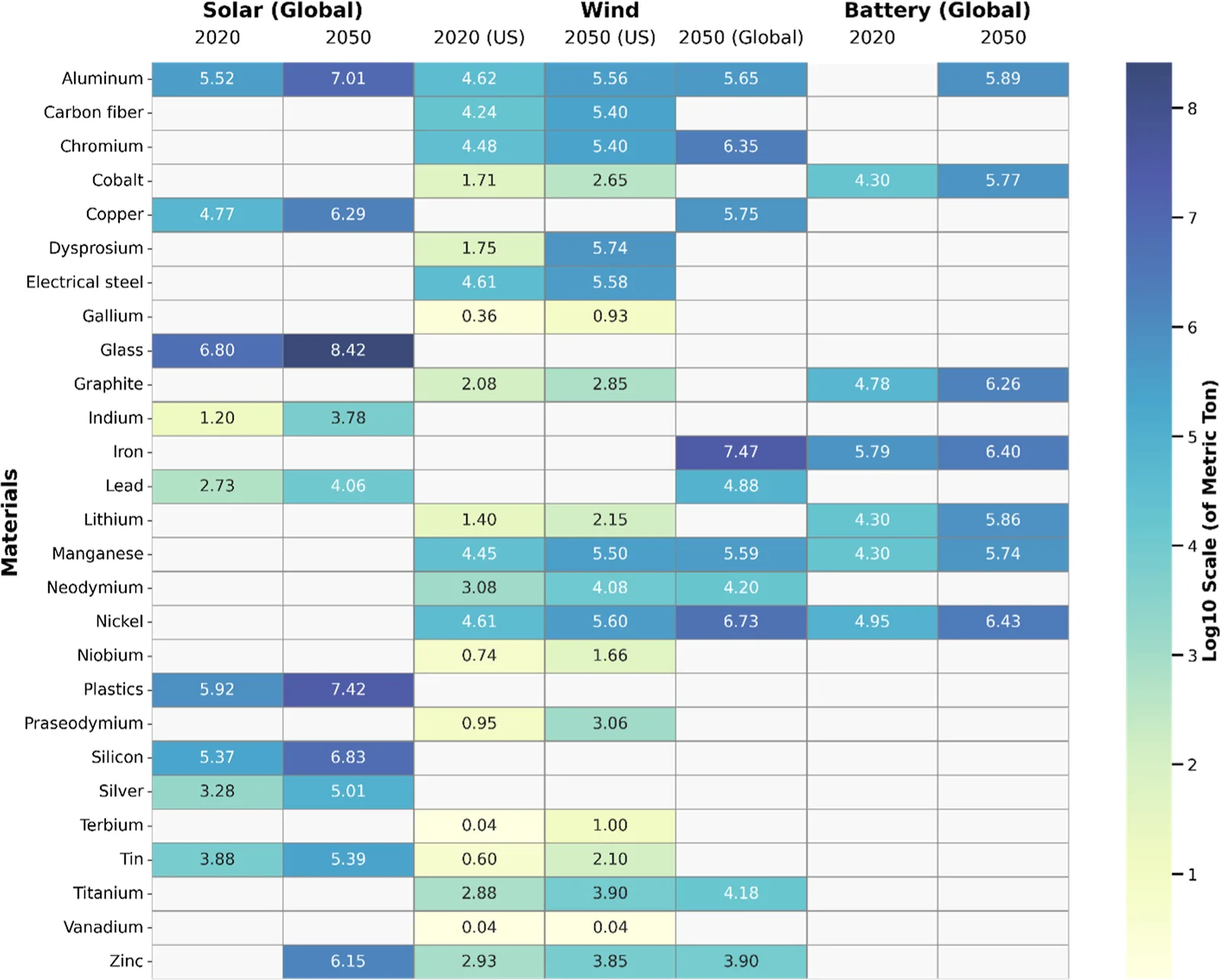
Selected materials demand (log scale) resulting from the development and deployment of renewable technologies. Raw data are provided in Table S2. Cells marked in light gray color indicate either no information or that the respective material is indeed not used much in the respective technology.

The escalating demand for raw materials will exacerbate mining, which is expected to intensify chemical releases to air, water,soil,^82^ and associated impacts on biodiversity.^83^ For example, production of every tonne of REE materials is estimated to lead to a chemical footprint of 13 kg of dust, 9600–12,000 m^3^ of waste gas containing hydrofluoric acid and sulfur dioxide, 75 m^3^ of acidic wastewater, and 1 tonne of radioactive waste residues.^84^ Mining only represents one aspect of the broader chemical footprint associated with the full life cycle of renewable energy infrastructure, as discussed in the previous section. The projected growth in material demand (Figure 3) also implies a corresponding increase in the magnitude and complexity of chemical flows across their life cycles. Although the chemical release potential of several life cycle stages remains insufficiently characterized, these releases can nonetheless be expected to scale in a manner similar to the projected raw material demands (Figure 3).

Overall, as deployment scales, the renewable infrastructure will not only generate larger volumes of chemical inputs and emissions during raw material extraction and manufacturing but also contribute to cumulative environmental loading over time. Considering the long operational lifetimes of renewable infrastructure, which range from years for batteries to decades for solar panels and wind turbines, the accumulation of materials in use represents a growing reservoir of embedded chemicals that may be released gradually during operation or more abruptly at EOL stage. This stock-flow dynamic highlights the importance of linking material demand, infrastructure growth, and chemical releases throughout the entire life cycle(s) to better understand long-term environmental exposure. As the chemical release potential of some life cycle stages is even less well-known, but can be expected to scale similarly as the raw material demands (Figure 3), addressing and understanding the entirety of embedded chemical impacts of renewable energy systems becomes an environmental urgency.

## Moving Forward: From Conventional Renewables to Truly Green Energy Technologies

In summary, while current renewable energy technologies offer substantial climate benefits, they may also carry significant chemical impacts across their entire life cycle, which challenges them being truly sustainable solutions. The design and optimization of all energy technologies inherently involve trade-offs across multiple environmental impact categories. Accordingly, more holistic and integrated approaches are needed to better identify, characterize, and manage these trade-offs when developing, redesigning, and implementing renewable technologies. At the same time, such comprehensive assessments fall beyond the scope of this Perspective article. Our goal here is to highlight the importance of more systematically considering chemical release, exposure pathways, and degradation products throughout the life cycle of renewable energy technologies.

As a next step, the broader renewable energy community may explore how best to integrate chemical impact considerations early into new technology development and identify and address chemical impact hotspots across existing technologies, along with other environmental impacts. This will help support the sustainable transition to renewable energy technologies with maximized environmental benefits and minimized trade-offs across impact categories. In this context, quantitative or semiquantitative approaches, such as emission thresholds, comparative toxicity metrics, and material substitution benchmarks,^85–87^ may serve as a useful starting point to support the assessment and prioritization of alternative technologies and materials.

With the anticipated large-scale deployment of renewable energy infrastructures, their embedded chemical footprint is likely to increase rapidly. Given the long operational lifespans of these systems, ranging from several years for batteries to multiple decades for solar panels and wind turbines, it is imperative to take swift and serious action to address the chemical footprint of renewable energy technologies to prevent and mitigate long-term environmental and health risks. This requires concerted efforts from all relevant stakeholders, including researchers, industry leaders, and policymakers. Crucially, a shift in mindset is needed: addressing the embedded chemical footprint of renewable energy technologies is not merely about mitigating environmental harm, it presents a strategic opportunity to ensure that the energy transition is both innovative and genuinely aligned with a safer and more sustainable future. Ultimately, addressing the chemical dimensions of renewable energy systems is not to challenge the importance of renewable deployment, but to help ensure that energy transition remains aligned with society’s goals of long-term sustainability.

Before such operationalization is feasible, the broad renewable energy community should first systematically examine the embedded chemical footprint throughout the life cycle of renewable energy technologies, starting with identifying the chemicals involved, characterizing exposure pathways, and assessing associated impacts. A key challenge for understanding the embedded chemical footprint of renewable energy systems is the limited knowledge of degradation products formed during operation and EOL processes. For example, offshore wind infrastructure can release transformation products from the degradation of coatings and corrosion protection systems, while battery recycling and thermal treatment processes can generate a range of secondary compounds, including fluorinated species, volatile organics, and other byproducts that are often not fully characterized. These substances may differ significantly from the original materials in terms of their properties, such as mobility, persistence, and toxicity, but are rarely included in conventional analyses. All this knowledge can then inform, e.g., what types of data are required to establish actionable thresholds, how uncertainties in degradation pathways or emissions during recycling should be addressed, and how increased transparency in material composition might be translated into effective regulatory metrics.

### Developing Innovative Renewable Energy Technologies

Most, if not all, current renewable energy technologies involve hazardous chemicals throughout their life cycles. Therefore, innovation aimed at developing renewable technologies that eliminate or minimize the use of hazardous substances is urgently needed. This is particularly critical for future, high-volume technologies such as PSCs or solid-state batteries (SSBs), where “lock-in” has not yet occurred.^88^ Early intervention in the design and development of these technologies presents a unique opportunity to embed safer chemical practices and avoid long-term environmental and health risks. SSBs, for example, are the next-generation battery technology due to their higher energy densities and greater safety than LIBs. Emerging SSBs include oxides, sulfides/thiophosphates/halides, polymers, and hybrid SSBs. It can be expected that SSBs contain diverse groups of fluorinated compounds, including PFASs, in their solid electrolytes and binders.^50^ To shift the trajectory toward safer innovation, the research community should begin by comprehensively mapping the embedded chemical footprints of various SSB types and prioritizing those with minimal hazardous chemical profiles for further development.

Additionally, PFASs serve as a compelling case to demonstrate the need for systemic approaches to managing chemical footprints throughout the energy transition. While substantial progress has been made in eliminating PFASs from food packaging, personal care products, and textiles, their replacement in energy technologies remains particularly challenging due to their exceptional performance and versatility. As many renewable technologies continue to undergo rapid research and development, now is an opportune moment for the community to critically reconsider their chemical and material design, particularly by prioritizing innovative PFAS-free chemistries that can deliver comparable functionality without long-term environment and health trade-offs. The ongoing operationalization of the EU’s Safe-and-Sustainable-by-Design (SSbD) framework may offer valuable tools to guide the development of renewable energy technologies with reduced chemical footprints.^89^ While some promising developments are already underway, broader interdisciplinary engagement will be essential to accelerate meaningful change. Importantly, addressing the embedded chemical footprint should not be viewed solely as means to reducing environmental harm, but as a strategic opportunity to foster innovation and ensure that the global energy transition is both safe and genuinely sustainable.

### In-Use Renewable Energy Infrastructure

In addition to addressing the chemical footprint of new renewable energy infrastructure prior to market entry, existing infrastructure, particularly those approaching their EOL stage, requires urgent attention. Without proper management, these infrastructures may continue to contribute to ecosystem and human exposure to hazardous chemicals, either embedded during production or generated through improper treatment and disposal. The shift to renewable energy infrastructures is driving a steep increase in minerals and materials demands, positioning the energy sector as a major force in global materials markets. Recycling can alleviate the pressure on primary material supply, yet current recovery practices remain limited for many energy transition materials such as lithium and rare earth elements. Scaling up recycling infrastructure could reduce raw materials demand by 13–35% over the long-term, while mitigating the mining-related environmental impacts.^80,90^ However, recycling practices should be designed to prevent the release of, and exposure to, other hazardous chemicals contained within EOL infrastructure.

The traditional fossil fuel industry operates predominantly on a linear value chain, characterized by a straightforward trajectory from raw material extraction, consumption, and ultimately disposal, which has come at significant environmental costs. In contrast, the renewable energy economy must move beyond this linear model. As renewable technologies and their infrastructures expand, their intensive material and chemical demands will require not only technological innovation but also systematic coordination across the entire value chain, including responsible sourcing, transparent material tracking to sustainable design and recovery. Given the long operational lifespan of renewable energy infrastructure, the associated chemical impacts may endure for decades, highlighting the urgent need to address the chemical footprints embedded throughout their life cycles. Addressing this gap demands traceable, accessible, and standardized information on material composition. At the European level, the Digital Product Passport (DPP) is being developed to embed material and chemical transparency throughout a product’s entire life cycle, with the battery passport serving as an early example. As batteries play a central role in enabling the clean energy transition, extending similar approaches to other renewable technologies could be transformative to facilitate circularity and reduce embedded chemical footprints in the energy sector. Ultimately, collaborative efforts among scientists, industry, and policymakers to incorporate chemical footprint thinking can help anticipate unintended consequences and amplify the voice to enable a true sustainable energy transition. We hope this Perspective initiates a deeper and more fundamental dialogue on how renewable energy infrastructure is developed, deployed, and disposed of.

## Supplementary Material



## Notes

Additional description of data and sources, figures and tables showing descriptive analysis of relevant data sets (PDF)

## References

[1] U.S. Energy Information Administration . Annual Energy Outlook 2023; Washington, DC, 2023. https://www.eia.gov/outlooks/aeo/(accessed 2024–09–28).

[2] Panthi G., Bajagain R., An Y. J., Jeong S. W. (2021). Leaching Potential
of Chemical Species from Real Perovskite and Silicon Solar Cells. Process Saf. Environ. Prot..

[3] Su P., Liu Y., Zhang J., Chen C., Yang B., Zhang C., Zhao X. (2020). Pb-Based Perovskite Solar Cells and
the Underlying Pollution behind
Clean Energy: Dynamic Leaching of Toxic Substances from Discarded
Perovskite Solar Cells. J. Phys. Chem. Lett..

[4] Guelfo J. L., Ferguson P. L., Beck J., Chernick M., Doria-Manzur A., Faught P. W., Flug T., Gray E. P., Jayasundara N., Knappe D. R. U., Joyce A. S., Meng P., Shojaei M. (2024). Lithium-Ion
Battery Components Are at the Nexus of Sustainable Energy and Environmental
Release of per- and Polyfluoroalkyl Substances. Nat. Commun..

[5] Bălan S.A. (2025). Confronting the Interconnection
of Chemical Pollution and Climate
Change. Environ. Innov. Soc. Transit..

[6] Amponsah N. Y., Troldborg M., Kington B., Aalders I., Hough R. L. (2014). Greenhouse
Gas Emissions from Renewable Energy Sources: A Review of Lifecycle
Considerations. Renew. Sustain. Energy Rev..

[7] Millstein D., O’Shaughnessy E., Wiser R. (2024). Climate and Air Quality Benefits
of Wind and Solar Generation in the United States from 2019 to 2022. Cell Rep. Sustain..

[8] Blumenthal J., Diamond M. L., Liu G., Wang Z. (2022). Introducing “Embedded
Toxicity”: A Necessary Metric for the Sound Management of Building
Materials. Environ. Sci. Technol..

[9] Shamoushaki M., Koh S. C. L. (2024). Comparative Life Cycle Assessment
of Integrated Renewable
Energy-Based Power Systems. Sci. Total Environ..

[10] Pehnt M. (2006). Dynamic Life
Cycle Assessment (LCA) of Renewable Energy Technologies. Renew. Energy.

[11] Varun, Bhat I. K., Prakash R. (2009). LCA of Renewable
Energy for Electricity Generation Systems A Review. Renew. Sustain. Energy Rev..

[12] Torres J. F., Petrakopoulou F. (2022). A Closer Look at the Environmental
Impact of Solar
and Wind Energy. Global Challenges.

[13] Rabaia M. K. H., Abdelkareem M. A., Sayed E. T., Elsaid K., Chae K. J., Wilberforce T., Olabi A. G. (2021). Environmental Impacts
of Solar Energy Systems: A Review. Sci. Total
Environ..

[14] Billen P., Leccisi E., Dastidar S., Li S., Lobaton L., Spatari S., Fafarman A. T., Fthenakis V. M., Baxter J. B. (2019). Comparative Evaluation of Lead Emissions and Toxicity
Potential in the Life Cycle of Lead Halide Perovskite Photovoltaics. Energy.

[15] Wang R., Huang T., Xue J., Tong J., Zhu K., Yang Y. (2021). Prospects for Metal
Halide Perovskite-Based Tandem Solar Cells. Nature Photonics.

[16] Jiang Y., Qiu L., Juarez-Perez E. J., Ono L. K., Hu Z., Liu Z., Wu Z., Meng L., Wang Q., Qi Y. (2019). Reduction
of Lead Leakage from Damaged Lead Halide Perovskite Solar Modules
Using Self-Healing Polymer-Based Encapsulation. Nat. Energy.

[17] Chen B., Fei C., Chen S., Gu H., Xiao X., Huang J. (2021). Recycling
Lead and Transparent Conductors from Perovskite Solar Modules. Nat. Commun..

[18] Walzberg J., Carpenter A., Heath G. A. (2021). Role of the Social Factors in Success
of Solar Photovoltaic Reuse and Recycle Programmes. Nat. Energy.

[19] Ibn-Mohammed T., Koh S. C. L., Reaney I. M., Acquaye A., Schileo G., Mustapha K. B., Greenough R. (2017). Perovskite
Solar Cells: An Integrated
Hybrid Lifecycle Assessment and Review in Comparison with Other Photovoltaic
Technologies. Renew. Sustain. Energy Rev..

[20] Hoang M. T., Yang Y., Pham N. D., Wang H. (2024). Ecofriendly Solution
Processing of Perovskite Solar Cells Using Water. Environ. Sci. Technol..

[21] Sherwood J., Albericio F., de la Torre B. G. N. (2024). N-Dimethyl Formamide European Restriction
Demands Solvent Substitution in Research and Development. ChemSusChem.

[22] Torrence C. E., Libby C. S., Nie W., Stein J. S. (2023). Environmental and
Health Risks of Perovskite Solar Modules: Case for Better Test Standards
and Risk Mitigation Solutions. iScience.

[23] Glüge J., Scheringer M., Cousins I. T., Dewitt J. C., Goldenman G., Herzke D., Lohmann R., Ng C. A., Trier X., Wang Z. (2020). An Overview of the Uses of Per- And
Polyfluoroalkyl Substances (PFAS). Environ.
Sci. Process. Impacts.

[24] Gao W., Yu L., Lin H., Meng L., Yu S., Li J., Lin Y., Zheng Y. (2024). A Review on the Impacts of Fluorinated Organic Additives
in Lithium Battery Industry an Emerging Source of per-and Polyfluoroalkyl
Substances. Crit. Rev. Environ. Sci. Technol..

[25] Rensmo A., Savvidou E. K., Cousins I. T., Hu X., Schellenberger S., Benskin J. P. (2023). Lithium-Ion Battery
Recycling: A Source of per- and
Polyfluoroalkyl Substances (PFAS) to the Environment?. Environ. Sci. Process. Impacts.

[26] Fenton S. E., Ducatman A., Boobis A., DeWitt J. C., Lau C., Ng C., Smith J. S., Roberts S. M. (2020). Per- and Polyfluoroalkyl Substance
Toxicity and Human Health Review: Current State of Knowledge and Strategies
for Informing Future Research. Environ. Toxicol.
Chem..

[27] Nain P., Anctil A. (2025). Per- and Polyfluoroalkyl
Substances (PFAS) in Solar
Photovoltaic Modules. Renew. Sustain. Energy
Rev..

[28] Chen P. H., Chen W. S., Lee C. H., Wu J. Y. (2023). Comprehensive Review
of Crystalline Silicon Solar Panel Recycling: From Historical Context
to Advanced Techniques. Sustainability.

[29] Prakash O., Tiwari S., Maiti P. (2022). Fluoropolymers and Their Nanohybrids
As Energy Materials: Application to Fuel Cells and Energy Harvesting. ACS Omega.

[30] Lohmann R., Cousins I. T., Dewitt J. C., Glüge J., Goldenman G., Herzke D., Lindstrom A. B., Miller M. F., Ng C. A., Patton S., Scheringer M., Trier X., Wang Z. (2020). Are Fluoropolymers Really of Low
Concern for Human and Environmental Health and Separate from Other
PFAS?. Environ. Sci. Technol..

[31] Organisation for Economic Co-operation and Development . Synthesis Report on Understanding Side-Chain Fluorinated Polymers and Their Life Cycle OECD Series on Risk Management No. 73; 2022. https://www.oecd.org/en/publications/synthesis-report-on-understanding-side-chain-fluorinated-polymers-and-their-life-cycle_e13559f7-en.html (accessed 2025–07–21).

[32] European Union . Commission Regulation (EU) 2024/2462 of 17 September 2024 Amending Annex XVII to Regulation (EC) No 1907/2006 of the European Parliament and of the Council as Regards Undecafluorohexanoic Acid (PFHxA), Its Salts and PFHxA-Related Substances; 2024. http://data.europa.eu/eli/reg/2024/2462/oj (accessed 2025–07–22).

[33] Skjolding, L. ; Olsson, M. ; Özkan, S. ; Mayer, P. ; Baun, A. Quantifying PFAS Release From Solar Panels During the Use Phase. In SETAC Europe 35th Annual Meeting, 2025.

[34] Dauchy X. (2023). Evidence of
Large-Scale Deposition of Airborne Emissions of per- and Polyfluoroalkyl
Substances (PFASs) near a Fluoropolymer Production Plant in an Urban
Area. Chemosphere.

[35] Prevedouros K., Cousins I. T., Buck R. C., Korzeniowski S. H. (2006). Sources,
Fate and Transport of Perfluorocarboxylates. Environ. Sci. Technol..

[36] Northeastern University . Studying Social, Scientific, and Political Factors of Per- and Polyfluoroalkyl Substances. the PFAS Project Lab. https://pfasproject.com/(accessed 2025–10–21).

[37] Wang Z., Cousins I., Scheringer M., Hungerbühler K. (2013). Fluorinated
Alternatives to Long-Chain Perfluoroalkyl Carboxylic Acids (PFCAs),
Perfluoroalkane Sulfonic Acids (PFSAs) and Their Potential Precursors. Environ. Int..

[38] Wang Z., Cousins I. T., Scheringer M., Hungerbuehler K. (2015). Hazard Assessment
of Fluorinated Alternatives to Long-Chain Perfluoroalkyl Acids (PFAAs)
and Their Precursors: Status Quo, Ongoing Challenges and Possible
Solutions. Environ. Int..

[39] Sworen J. C., Morken P. A., Smith A. P., Boyle J. E., Cervantes
Garcia M. D., Kramer J., Wadsley M. P., Davis M. C. (2024). Interrogation
of a Fluoropolymer Dispersion Manufactured with a Non-Fluorinated
Polymerization Aid for Targeted and Non-Targeted Fluorinated Residuals
by Liquid Chromatography High Resolution Mass Spectrometry. J. Chromatogr. A.

[40] Dalmijn J., Glüge J., Scheringer M., Cousins I. T. (2024). Emission Inventory
of PFASs and Other Fluorinated Organic Substances for the Fluoropolymer
Production Industry in Europe. Environ. Sci.
Process. Impacts.

[41] Dalmijn, J. Emissions of Per- and Polyfluoroalkyl Substances (PFAS) by Fluoropolymer Production Plants. Doctor of Philosophy; Stockholm University: Sweden, 2025.

[42] Newton S., McMahen R., Stoeckel J. A., Chislock M., Lindstrom A., Strynar M. (2017). Novel Polyfluorinated Compounds Identified
Using High
Resolution Mass Spectrometry Downstream of Manufacturing Facilities
near Decatur, Alabama. Environ. Sci. Technol..

[43] Stanley K. M., Say D., Mühle J., Harth C. M., Krummel P. B., Young D., O’Doherty S. J., Salameh P. K., Simmonds P. G., Weiss R. F., Prinn R. G., Fraser P. J., Rigby M. (2020). Increase in Global
Emissions of HFC-23 despite near-Total Expected Reductions. Nat. Commun..

[44] Montzka, S. A. ; Burkholder, J. B. ; Carpenter, L. J. ; Fahey, D. W. ; Jucks, K. W. ; Safari, B. Montreal Protocol on Substances that Deplete the Ozone Layer, 2024.

[45] Zatoń M., Rozière J., Jones D. J. (2017). Current Understanding of Chemical
Degradation Mechanisms of Perfluorosulfonic Acid Membranes and Their
Mitigation Strategies: A Review. Sustain. Energy
Fuels.

[46] Ellis D. A., Mabury S. A., Martin J. W., Muir D. C. G. (2001). Thermolysis of
®Uoropolymers as a Potential Source of Halogenated Organic Acids
in the Environment. Nature.

[47] Ellis D. A., Martin J. W., Muir D. C. G., Mabury S. A. (2003). The Use of 19F NMR
and Mass Spectrometry for the Elucidation of Novel Fluorinated Acids
and Atmospheric Fluoroacid Precursors Evolved in the Thermolysis of
Fluoropolymers. Analyst.

[48] Feng M., Qu R., Wei Z., Wang L., Sun P., Wang Z. (2015). Characterization
of the Thermolysis Products of Nafion Membrane: A Potential Source
of Perfluorinated Compounds in the Environment. Sci. Rep..

[49] Cui J., Guo J., Zhai Z., Zhang J. (2019). The Contribution of Fluoropolymer
Thermolysis to Trifluoroacetic Acid (TFA) in Environmental Media. Chemosphere.

[50] Tawalbeh T., Rangarajan S., Liu H., Wang Y. (2025). Fluorinated Substances
in Lithium-Ion Batteries and Solid State Batteries: Recycling Challenges
and Environmental Impacts. J. Power Sources.

[51] Longendyke G. K., Katel S., Wang Y. (2022). PFAS Fate
and Destruction Mechanisms
during Thermal Treatment: A Comprehensive Review. Environ. Sci.: Processes Impacts.

[52] Rodgers M. P., Bonville L. J., Kunz H. R., Slattery D. K., Fenton J. M. (2012). Fuel Cell
Perfluorinated Sulfonic Acid Membrane Degradation Correlating Accelerated
Stress Testing and Lifetime. Chem. Rev..

[53] Alipour
Moghaddam J., Parnian M. J., Rowshanzamir S. (2018). Preparation,
Characterization, and Electrochemical Properties Investigation of
Recycled Proton Exchange Membrane for Fuel Cell Applications. Energy.

[54] Behera, A. Fuel Cells Recycling. In Nanotechnology in Fuel Cells; Elsevier, 2022; pp 361–373.

[55] Wu B., Zhao M., Shi W., Liu W., Liu J., Xing D., Yao Y., Hou Z., Ming P., Gu J., Zou Z. (2014). The Degradation Study of Nafion/PTFE Composite Membrane
in PEM Fuel Cell under Accelerated Stress Tests. Int. J. Hydrogen Energy.

[56] Williams G. D. Z., Vengosh A. (2025). Quality of Wastewater from Lithium-Brine Mining. Environ. Sci. Technol. Lett..

[57] Meng L., Song B., Lu Y., Lv K., Gao W., Wang Y., Jiang G. (2021). The Occurrence of Per-
and Polyfluoroalkyl
Substances (PFASs) in Fluoropolymer Raw Materials and Products Made
in China. J. Environ. Sci. (Beijing, China).

[58] Christensen B. T., Calkins M. M. (2023). Occupational Exposure
to Per- and Polyfluoroalkyl Substances:
A Scope Review of the Literature from 1980–2021. J. Expo. Sci. Environ. Epidemiol..

[59] Quant M., Willstrand O., Mallin T., Hynynen J. (2023). Ecotoxicity
Evaluation
of Fire-Extinguishing Water from Large-Scale Battery and Battery Electric
Vehicle Fire Tests. Environ. Sci. Technol..

[60] Mrozik W., Rajaeifar M. A., Heidrich O., Christensen P. (2021). Environmental
Impacts, Pollution Sources and Pathways of Spent Lithium-Ion Batteries. Energy Environ. Sci..

[61] Willstrand O., Quant M., Hynynen J. (2025). Contaminations from Lithium-Ion Battery
Fires Per- and Polyfluoroalkyl Substances (PFAS) in Soot. Fire Technol..

[62] Lombardo G., Ebin B., Steenari B. M., Alemrajabi M., Karlsson I., Petranikova M. (2021). Comparison
of the Effects of Incineration,
Vacuum Pyrolysis and Dynamic Pyrolysis on the Composition of NMC-Lithium
Battery Cathode-Material Production Scraps and Separation of the Current
Collector. Resour., Conserv. Recycl..

[63] Jie Y., Yang S., Li Y., Hu F., Zhao D., Chang D., Lai Y., Chen Y. (2020). Waste Organic
Compounds
Thermal Treatment and Valuable Cathode Materials Recovery from Spent
LiFePO4 Batteries by Vacuum Pyrolysis. ACS Sustain.
Chem. Eng..

[64] Xu Y., Song D., Li L., An C., Wang Y., Jiao L., Yuan H. A. (2014). Simple
Solvent Method for the Recovery
of LixCoO2 and Its Applications in Alkaline Rechargeable Batteries. J. Power Sources.

[65] Al-Asheh S., Aidan A., Allawi T., Hammoud F., Al Ali H., Al Khamiri M. (2023). Treatment
and Recycling of Spent Lithium-Based Batteries:
A Review. J. Mater. Cycles Waste Manage..

[66] Du K., Ang E. H., Wu X., Liu Y. (2022). Progresses in Sustainable
Recycling Technology of Spent Lithium-Ion Batteries. Energy and Environmental Materials.

[67] Xu C., Dai Q., Gaines L., Hu M., Tukker A., Steubing B. (2020). Future Material
Demand for Automotive Lithium-Based Batteries. Commun. Mater..

[68] Wu J., Zheng M., Liu T., Wang Y., Liu Y., Nai J., Zhang L., Zhang S., Tao X. (2023). Direct Recovery: A
Sustainable Recycling Technology for Spent Lithium-Ion Battery. Energy Storage Materials.

[69] Hengstmann E., Corella P. Z., Alter K., Belzunce-Segarra M. J., Booth A. M., Castro-Jiménez J., Czerner N., De Cauwer K., Deviller G., Gomiero A., Goseberg N., Hasenbein S., Kirchgeorg T., Mason C., Pape W., Parmentier K., Plaß A., Pröfrock D., Sarhadi A., Vanavermaete D., van der Molen J., Vinagre P. A., Wood D., Weinberg I., Windt C., Zonderman A., Kenyon J., De Witte B. (2025). Chemical Emissions
from Offshore Wind Farms: From Identification to Challenges in Impact
Assessment and Regulation. Mar. Pollut. Bull..

[70] Zapata Corella, P. ; Hengstmann, E. ; Castro-Jiménez, J. ; Deviller, G. ; Vanavermaete, D. ; Plaß, A. ; De Witte, B. Literature Study on Chemical Emissions from Offshore Wind Farms_Project Anemoi. Zenodo 2025.

[71] Luft A., Bröder K., Kunkel U., Schulz M., Dietrich C., Baier R., Heininger P., Ternes T. A. (2017). Nontarget Analysis
via LC-QTOF-MS to Assess the Release of Organic Substances from Polyurethane
Coating. Environ. Sci. Technol..

[72] Zapata Corella, P. ; Hengstmann, E. ; Castro-Jiménez, J. ; Deviller, G. ; Vanavermaete, D. ; Plaß, A. ; De Witte, B. Literature Study on Chemical Emissions from Offshore Wind Farms_Project Anemoi, 2025

[73] Watson G. J., Banfield G., Watson S. C. L., Beaumont N. J., Hodkin A. (2025). Offshore Wind
Energy: Assessing Trace Element Inputs and the Risks for Co-Location
of Aquaculture. NPJ. Ocean Sustainability.

[74] Watson S. C. L., Szostek C. L., Edwards-Jones A., Wills B., Watson G. J., Beaumont N. (2025). Assessing, Monitoring
and Mitigating the Effects of
Offshore Wind Farms on Biodiversity. Nat. Rev.
Biodivers..

[75] Morales-Simfors N., Bundschuh J. (2022). Arsenic-Rich
Geothermal Fluids as Environmentally Hazardous
Materials – A Global Assessment. Sci.
Total Environ..

[76] Mott A., Baba A., Hadi Mosleh M., Ökten H. E., Babaei M., Gören A. Y., Feng C., Recepoğlu Y. K., Uzelli T., Uytun H., Morata D., Yüksel A., Sedighi M. (2022). Boron in Geothermal
Energy: Sources, Environmental
Impacts, and Management in Geothermal Fluid. Renew. Sustain. Energy Rev..

[77] Robertson D. E., Crecelius E. A., Fruchter J. S., Ludwick J. D. (1977). Mercury Emissions
from Geothermal Power Plants. Science.

[78] International Energy Agency . The Role of Critical World Energy Outlook Special Report Minerals in Clean Energy Transitions; 2022. www.iea.org/t&c/.

[79] International Energy Agency The Role of Critical Minerals in Clean Energy Transitions; Paris, 2021.

[80] Xu C., Isabella O., Vogt M. R. (2024). Future Material Demand for Global
Silicon-Based PV Modules under Net-Zero Emissions Target until 2050. Resour., Conserv. Recycl..

[81] Cooperman, A. ; Eberle, A. ; Hettinger, D. ; Marquis, M. ; Smith, B. ; Tusing, R. F. ; Walzberg, J. Renewable Energy Materials Properties Database: Summary, 2020; . www.nrel.gov/publications.

[82] Pecina V., Juřička D., Hedbávný J., Klimánek M., Kynický J., Brtnický M., Komendová R. (2023). The Impacts
of Mining on Soil Pollution with Metal­(Loid)­s
in Resource-Rich Mongolia. Sci. Rep..

[83] Sonter L. J., Dade M. C., Watson J. E. M., Valenta R. K. (2020). Renewable Energy
Production Will Exacerbate Mining Threats to Biodiversity. Nat. Commun..

[84] Konsult, K. Health and Safety Issues in REE Mining and Processing an Internal EURARE Guidance Report, 2014.

[85] Fantke P., Aurisano N., Bare J., Backhaus T., Bulle C., Chapman P. M., De Zwart D., Dwyer R., Ernstoff A., Golsteijn L., Holmquist H., Jolliet O., McKone T. E., Owsianiak M., Peijnenburg W., Posthuma L., Roos S., Saouter E., Schowanek D., van Straalen N. M., Vijver M. G., Hauschild M. (2018). Toward Harmonizing
Ecotoxicity Characterization
in Life Cycle Impact Assessment. Environ. Toxicol.
Chem..

[86] Tickner J.
A., Schifano J. N., Blake A., Rudisill C., Mulvihill M. J. (2015). Advancing
Safer Alternatives Through Functional Substitution. Environ. Sci. Technol..

[87] Rosenbaum R. K., Bachmann T. M., Gold L. S., Huijbregts M. A. J., Jolliet O., Juraske R., Koehler A., Larsen H. F., MacLeod M., Margni M., McKone T. E., Payet J., Schuhmacher M., Van De Meent D., Hauschild M. Z. (2008). USEtox
- The UNEP-SETAC Toxicity Model: Recommended Characterisation Factors
for Human Toxicity and Freshwater Ecotoxicity in Life Cycle Impact
Assessment. Int. J. Life Cycle Assess..

[88] Blumenthal J., Diamond M. L., Hoffmann M., Wang Z. (2022). Time to Break the “Lock-In”
Impediments to Chemicals Management. Environ.
Sci. Technol..

[89] van
Dijk J., Sharma A., Nowack B., Wang Z., Scheringer M. (2025). From Ambition
to Action: Navigating Obstacles and Opportunities of “Safe
and Sustainable by Design.”. Environ.
Sci. Technol..

[90] Liang Y., Kleijn R., van der
Voet E. (2023). Increase in Demand for Critical Materials
under IEA Net-Zero Emission by 2050 Scenario. Appl. Energy.

