# Supplementary material for: Deciphering the Chemical Footprint of Renewables
Source: Environ Sci Technol. 2026 Aug 4;60(32):22144–55. doi: 10.1021/acs.est.5c17593 (PMC13492264; doi:10.1021/acs.est.5c17593)
Supplement: Supplementary file 1 [file es5c17593_si_001.pdf]

Supporting Information

for

Deciphering the Chemical Footprint of Renewables

Tasneem Tawalbeh,[1] Zhanyun Wang,[*] Kathrin Fenner,[*] Yuxin Wang[*]

[1] *Tasneem Tawalbeh, Systems Science and Industrial Engineering, Binghamton University, 440 Vestal Pkwy E, Binghamton, NY, 13902 USA, ttawalb1@binghamton.edu*

** Corresponding author, Zhanyun Wang, Department of Green Technology & Danish Institute for Advanced Study (DIAS), University of Southern Denmark, 5230 Odense, Denmark; Empa–Swiss Federal Laboratories for Materials Science and Technology, Technology and Society Laboratory, 9014 St. Gallen, Switzerland; National Centre of Competence in Research (NCCR) Catalysis, ETH Zürich, 8093 Zürich, Switzerland; zwa@igt.sdu.dk*

** Corresponding author, Kathrin Fenner, Department of Environmental Chemistry, Swiss Federal Institute of Aquatic Science and Technology (Eawag), Dübendorf 8600, Switzerland; Department of Chemistry, University of Zürich, Zürich 8057, Switzerland; National Centre of Competence in Research (NCCR) Catalysis, ETH Zürich, 8093 Zürich, Switzerland; kathrin.fenner@chem.uzh.ch*

** Corresponding author, Yuxin Wang, Systems Science and Industrial Engineering, Binghamton University, 440 Vestal Pkwy E, Binghamton, NY, 13902, USA, yuxinw@binghamton.edu*

This document contains 7 pages with 3 pages of text, 2 tables, and 2 figures.

Text 1.1 Side-chain fluorinated polymers

Side-chain fluorinated polymers such as (heptadecafluoro-1,1,2,2-tetra-hydrodecyl) triethoxysilane are used in solar cells to improve their performance, stability, hydrophobic characteristics, and efficiency.[1] Other possible PFAS use in crystalline silicon solar modules include fluorinated ethylene propylene (FEP) and trichloro(1H, 1H, 2H, 2H -perfluorooctyl)silane as anti-soiling and self-cleaning coatings.[2] These side-chain fluorinated polymers may contain low-molecular-weight PFASs as impurities, or break down and form low-molecular-weight PFASs, and thus release them into the environment along the life cycle.[3] Indeed, low levels of perfluorohexanoic acid (PFHxA), which are restricted in the EU.[4] have been detected on eight bifacial solar panels from different producers during their use phase.[5]

Table S1: Data and sources used to create Figure 1: Electricity generation from renewable technologies in 2022 and great expansions projected for 2050 globally. The unit is Billion kWh.

Technology	Worldwide [6]		US [7]		Western Europe [8]	
	2022	2050	2022	2050	2022	2050
**Hydro**	4320	5583	276	285	621	754
**Wind**	1967	5805	440	1218	474	962
**Geothermal**	67	254	16	37	22	57
**Solar**	1421	8521	205	1828	209	849
**Other**	669	903	67	57	323	614

Table S2: Original data in metering ton used to create Figure 3: Selected materials demand (log scale) resulting from the development and deployment of renewable technologies.[9–13] Empty cells indicate either no information or that the respective material is indeed not used much in the respective.

Key Materials	Solar (Global)		Wind			Battery (Global)	
	2020	2050	2020 (US)	2050 (US)	2050 (Global)	2020	2050
**Aluminum**	333000	10200000	41500	360000	446000		770000
**Carbon fiber**			17500	250000			
**Chromium**			30500	250000	2263000		
**Cobalt**			50	450		20000	590000
**Cooper**	59000	1950000			563000		
**Dysprosium**			55	550000			
**Electrical steel**			40500	380000			
**Gallium**			1.3	7.5			
**Glass**	6310000	262000000					
**Graphite**			120	700		60000	1810000
**Indium**	14.7	6070					
**Iron**					29215000	612000	2500000
**Lead**	537	11500			75000		
**Lithium**			24	140		20000	720000
**Manganese**			28500	315000	388000	20000	550000
**Neodymium**			1200	12000	16000		
**Nickel**			41000	395000	5339000	90000	2680000
**Niobium**			4.5	45			
**Plastics**	835000	26400000					
**Praseodymium**			8	1150			
**Silicon**	237000	6830000					

**Silver**	1890	103000					
**Terbium**			<0.1	9			
**Tin**	7520	248000	3	125			
**Titanium**			750	8000	15000		
**Vanadium**			<0.1	<0.1			
**Zinc**		1413000	850	7000	8000		

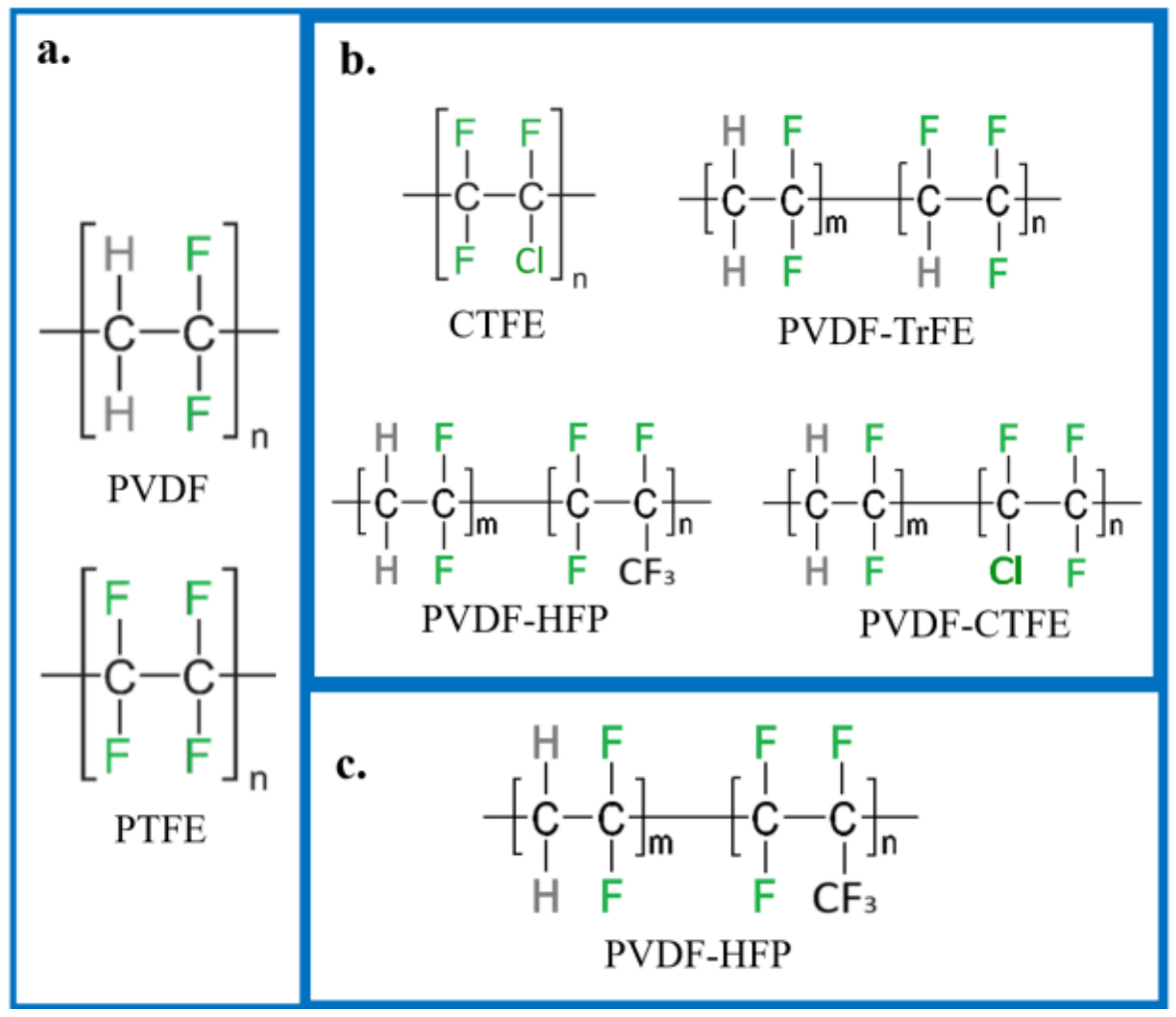

Figure S1: Fluoropolymers and copolymers used in: (a) fuel cells, LIBs, and SSB,[14,15] (b) fuel cells,[14] and (c) SSB.[16]

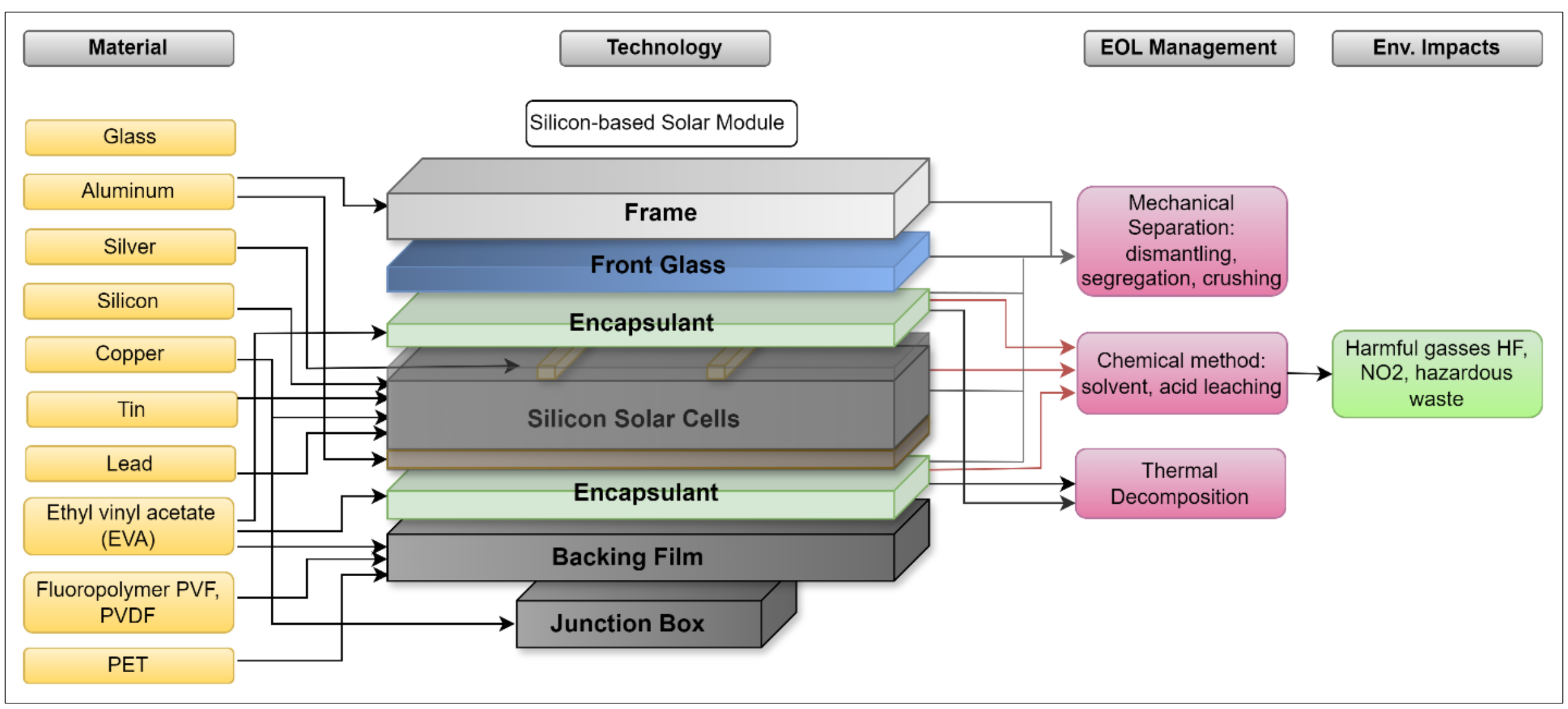

Figure S2: The metal and recycling methods applied in silicon-based solar module.[17,18]

References

1. Han HC, Tseng CA, Du CY, et al. Enhancing efficiency with fluorinated interlayers in small molecule organic solar cells. *J Mater Chem*. 2012;22(43):22899-22905. doi:10.1039/c2jm34091g

2. Nain P, Anctil A. Per- and polyfluoroalkyl substances (PFAS) in solar photovoltaic modules. *Renewable and Sustainable Energy Reviews*. 2025;215. doi:10.1016/j.rser.2025.115562

3. OECD. *Synthesis Report on Understanding Side-Chain Fluorinated Polymers and Their Life Cycle OECD Series on Risk Management No. 73*.; 2022. Accessed July 21, 2025. https://www.oecd.org/en/publications/synthesis-report-on-understanding-side-chain-fluorinated-polymers-and-their-life-cycle_e13559f7-en.html

4. European Union. *Commission Regulation (EU) 2024/2462 of 17 September 2024 Amending Annex XVII to Regulation (EC) No 1907/2006 of the European Parliament and of the Council as Regards Undecafluorohexanoic Acid (PFHxA), Its Salts and PFHxA-Related Substances*.; 2024. Accessed July 22, 2025. http://data.europa.eu/eli/reg/2024/2462/oj

5. Skjolding L, Olsson M, Özkan S, Mayer P, Baun A. Quantifying PFAS Release From Solar Panels During the Use Phase. In: *SETAC Europe 35th Annual Meeting* . 2025.

6. EIA. *Annual Energy Outlook - Electricity Installed Generating Capacity: World, Reference Case*.; 2023. Accessed September 30, 2024. https://www.eia.gov/outlooks/ieo/data/pdf/E_cap_E1.cap_r_230822.081459.pdf

7. EIA. *Annual Energy Outlook - Electricity Generation: United States, Reference Case*.; 2023. Accessed September 30, 2024. file:///C:/Users/tasst/Desktop/Research%20Projects/Renewable%20Energy/Perspective%20Paper/08252024_PerspectivePaper_1stMEeting_TT/energy%20grouwth%20table%20-%20USA.pdf

8. EIA. *Annual Energy Outlook - Electricity Generation: Western Europe, Reference Case*.; 2023. Accessed September 30, 2024. https://www.eia.gov/outlooks/ieo/data/pdf/E_gen_E9.gen_r_230822.081459.pdf

9. Eberle A, Cooperman A, Walzberg J, et al. *Materials Used in U.S. Wind Energy Technologies: Quantities and Availability for Two Future Scenarios*.; 2050. www.nrel.gov/publications.

10. Lokanc M, Eggert R, Redlinger M. *The Availability of Indium: The Present, Medium Term, and Long Term*.; 2015. www.nrel.gov/publications.

11. Maisel F, Neef C, Marscheider-Weidemann F, Nissen NF. A forecast on future raw material demand and recycling potential of lithium-ion batteries in electric vehicles. *Resour Conserv Recycl*. 2023;192. doi:10.1016/j.resconrec.2023.106920

12. Xu C, Dai Q, Gaines L, Hu M, Tukker A, Steubing B. Future material demand for automotive lithium-based batteries. *Commun Mater*. 2020;1(1). doi:10.1038/s43246-020-00095-x

13. Flanagan D. *Mineral Commodity Summaries (Copper)*.; 2024.

14. Prakash O, Tiwari S, Maiti P. Fluoropolymers and Their Nanohybrids As Energy Materials: Application to Fuel Cells and Energy Harvesting. *ACS Omega.American Chemical Society*. 2022;7(39):34718-34740. doi:10.1021/acsomega.2c04774

15. Rensmo A, Savvidou EK, Cousins IT, Hu X, Schellenberger S, Benskin JP. Lithium-ion battery recycling: a source of per- and polyfluoroalkyl substances (PFAS) to the environment? *Environ Sci Process Impacts.Royal Society of Chemistry*. 2023;25(6):1015-1030. doi:10.1039/d2em00511e

16. Wang TW, Liu T, Sun H. Direct Recycling for Advancing Sustainable Battery Solutions. *Mater Today Energy*. 2023;38. doi:10.1016/j.mtener.2023.101434

17. Chen PH, Chen WS, Lee CH, Wu JY. Comprehensive Review of Crystalline Silicon Solar Panel Recycling: From Historical Context to Advanced Techniques. *Sustainability (Switzerland).Multidisciplinary Digital Publishing Institute (MDPI)*. 2024;16(1). doi:10.3390/su16010060

18. Drabczyk K, Panek P. *Silicon-Based Solar Cells Characteristics and Production Processes*. Second Edition.; 2012. www.printpap.pl
